# EZH2 Promotes T Follicular Helper Cell Differentiation Through Enhancing STAT3 Phosphorylation in Patients With Primary Sjögren’s Syndrome

**DOI:** 10.3389/fimmu.2022.922871

**Published:** 2022-06-20

**Authors:** Chengmei He, Yanlei Yang, Zhilei Chen, Suying Liu, Taibiao Lyu, Liuting Zeng, Li Wang, Yongzhe Li, Mu Wang, Hua Chen, Fengchun Zhang

**Affiliations:** ^1^ Department of Rheumatology and Clinical Immunology, Peking Union Medical College Hospital, Peking Union Medical College, Chinese Academy of Medical Sciences, Key Laboratory of Rheumatology and Clinical Immunology, Ministry of Education, National Clinical Research Center for Dermatologic and Immunologic Diseases, Beijing, China; ^2^ Medical Science Research Centre, Peking Union Medical College Hospital, Chinese Academy of Medical Sciences and Peking Union Medical College, Beijing, China; ^3^ Department of Clinical Laboratory, Peking Union Medical College Hospital, Chinese Academy of Medical Science and Peking Union Medical College, Beijing, China; ^4^ Department of Stomatology, Peking Union Medical College Hospital, Chinese Academy of Medical Sciences & Peking Union Medical College, Beijing, China

**Keywords:** primary Sjögren’s syndrome, T follicular helper cell, enhancer zeste homolog 2 (EZH2), STAT3, T cell

## Abstract

**Objectives:**

Enhancer of zeste homolog 2 (EZH2) is an epigenetic regulator that plays an essential role in immune system development and autoimmune diseases. This study aimed to characterize the role of EZH2 in the pathogenesis of primary Sjögren’s syndrome (pSS).

**Methods:**

We analyzed EZH2 expression in two transcriptomic datasets of peripheral blood mononuclear cells (PBMCs) from pSS patients and healthy controls. We measured EZH2 expression in CD4^+^ T cells, CD8^+^ T cells, and CD19^+^ B cells from pSS patients and healthy controls and correlated EZH2 expression with clinical parameters. We also examined the activation, proliferation, and T-cell differentiation of CD4^+^ T cells using the EZH2 inhibitor GSK126, EZH2 siRNA, and EZH2-expressing vector. We further examined the STAT3 signaling pathway after EZH2 inhibition and detected Tfh differentiation in EZH2-overexpressed CD4^+^ T cells with STAT3 knocked down.

**Results:**

EZH2 was upregulated in GSE164885 and GSE48378. EZH2 expression was higher in pSS CD4^+^ and CD8+ T cells, and EZH2 expression in circulating pSS CD4^+^ T cells was positively correlated with IgG, IgA, ESR, RF, and the circulating Tfh population. EZH2 inhibition and silencing EZH2 suppressed activation, proliferation, and Tfh differentiation. Furthermore, overexpressing EZH2 promoted activation, proliferation, and Tfh differentiation in CD4^+^ T cells. EZH2 inhibition attenuated STAT3 phosphorylation in CD4^+^ T cells. STAT3 knockdown abrogated EZH2-promoted Tfh differentiation.

**Conclusions:**

EZH2 expression was abnormally elevated in pSS CD4^+^ T cells, which facilitated Tfh differentiation of CD4^+^ T cells by enhancing STAT3 phosphorylation. EZH2 promotes Tfh differentiation and might be implicated in pSS pathogenesis.

## Introduction

Primary Sjögren’s syndrome (pSS) is a systemic autoimmune disease characterized by lymphocytic infiltration of exocrine glands. pSS patients typically present with a triad of symptoms, including dry mouth and eyes, fatigue, and pain (1). In addition, about 40% of pSS patients suffer from systemic complications. However, the pathogenesis of pSS remains elusive, and no targeted therapy is currently available for pSS (2).

Abnormal activation of salivary gland epithelial cells induces persistent activation of the innate and adaptive immune systems, which is the key mechanism of pSS (3). CD4^+^ T cells are critically involved in the development of pSS as they form a large proportion of lymphocytes infiltrated into the exocrine glands, especially at an earlier stage of the disease. CD4^+^ T cells produce pro-inflammatory cytokines, leading to lymphocyte infiltration, B-cell activation, and tissue damage in pSS (4). CD4^+^ T cells are classified into several specialized subsets, namely, Th1, Th2, Th17, regulatory T (Treg), and T follicular helper (Tfh) cells. Maehara et al. reported higher expression of all Th subset-related cytokines, including interferon-γ (IFN-γ), interleukin-4 (IL-4), IL-17, IL-10, IL-21 in the labial salivary gland (LSG) of pSS. Moreover, Th2-related IL-4 and Tfh-related Bcl-6 were closely associated with lymphocyte infiltration in the LSG of pSS (5). Besides, the circulating Th17 cell number is increased in pSS, especially in patients with moderate to high disease activity (6).

Tfh cells are CD4^+^ T helper cell subsets with the expression of follicular homing C-X-C chemokine receptor type 5 (CXCR5), co-inhibitory molecule programmed death-1 (PD-1), transcription factor B-cell lymphoma 6 (Bcl6), inducible T-cell costimulator (ICOS), and the B cell helper cytokine IL-21 and play a critical role in assisting the humoral response mainly through IL-21, CD40 ligand (CD40L), and ICOS signaling (7). Dysregulated Tfh cells have been observed in systemic lupus erythematosus (SLE), rheumatoid arthritis (RA), and pSS (8). The number of Tfh cells is increased in the peripheral blood and LSG of pSS patients and positively correlates with the EULAR Sjögren’s syndrome disease activity index (ESSDAI) (9–12). Besides, circulating Tfh cells decreased after treatment in pSS patients (13–15). Tfh cells are also indispensable in the experimental Sjögren’s syndrome (ESS) model (16).

Enhancer of zeste homolog 2 (EZH2) is an enzymatic subunit of polycomb-repressive complex 2 (PRC2) that catalyzes the trimethylation of histone H3 lysine 27 (H3K27me3) and induces epigenetic gene silencing and transcriptional suppression (17). Besides, EZH2 methylate non-histone proteins, binds with transcription factors and activates transcription (18). EZH2 is essential for regulating critical biological activities such as X-chromosome inactivation, stem cell plasticity, and immune system development (17, 19). Evidence suggests that EZH2 is implicated in the pathogenesis of autoimmune diseases, including SLE, RA, and psoriasis (PsA) (20). EZH2 is overexpressed in CD4^+^ T and CD19^+^ B cells of lupus patients and promotes the adhesion, migration, extravasation of CD4^+^ T cells, and plasmablast differentiation (21, 22). EZH2 is downregulated in circulating CD4^+^ T cells and contributes to Treg deficiency in RA (23). EZH2 is upregulated in skin lesions of PsA patients and promotes proinflammatory cytokine production by keratinocytes (24). However, the function of EZH2 has not yet been explored in pSS.

In this study, we aim to identify the role of EZH2 in the pathogenesis of pSS, focus on the regulation of Tfh differentiation by EZH2, provide new insight into the mechanism of pSS pathogenesis, and propose a potential therapeutic target for pSS.

## Methods

### Subjects

pSS patients who fulfilled the classification criteria of the 2002 American European Consensus Group (25) and sex- and age-matched healthy controls (HCs) were enrolled as indicated (**Table S1**). Labial salivary gland samples were obtained from four pSS patients and four xerostomia patients who did not fulfill pSS criteria as the non-SS xerostomia controls. This study was approved by the ethics committee of Peking Union Medical College Hospital (S-K2023), and all participants provided written informed consent.

### Analysis of Transcriptomic Data of pSS Patients

The transcriptome data of peripheral blood mononuclear cells (PBMCs) from pSS patients and HCs were downloaded from the Gene Expression Omnibus. Log2 RMA-normalized signal intensity was used for microarray data analysis and counts were used for RNA-seq data. The upregulated genes were determined using a cut-off threshold of p-value of <0.05 and a fold change of >1.5, and the upregulated genes from two datasets were intersected.

### T-Cell Isolation, Activation, and Differentiation

PBMCs were isolated from pSS patients and HCs using Ficoll–Paque gradient centrifugation. CD4^+^CD45RA^+^ naïve T cells were purified from PBMCs using Naïve CD4^+^ T-Cell Isolation Kit II (Miltenyi Biotec, Germany) according to the instructions of the manufacturer, with a purity of more than 95% by flow cytometry. Naïve CD4^+^ T cells were activated with anti-CD3 (5 µg/ml, BD Bioscience) and anti-CD28 (5 µg/ml, BD Bioscience). For the T-cell differentiation, IL-12 (10 ng/ml, R&D systems) plus anti-IL4 (10 µg/ml, PeproTech), IL-4 (2 ng/ml, PeproTech) plus anti-IFNγ(10 µg/ml, BD Bioscience), anti-IL4 (10 µg/ml) plus anti-IFNγ(10 µg/ml) plus TGF-β(5 ng/ml, R&D systems) plus IL-1β(12.5 ng/ml, PeproTech) plus IL-6 (25 ng/ml, PeproTech) plus IL-23 (25 ng/ml, PeproTech), TGF-β1 (5 ng/ml, R&D systems) plus IL-12 (1 ng/ml, R&D systems), or IL-2 (5 ng/ml, R&D systems) plus TGF-β (5 ng/ml, R&D systems) were supplemented for Th1, Th2, Th17, Tfh or Treg differentiation, respectively. T cells were incubated in RPMI-1640 medium (Thermo Fisher) supplemented with 10% fetal bovine serum (FBS, Thermo Fisher) and at 37°C, 5% CO2 for 3–5 days.

### T-Cell Transfection

Naïve CD4^+^ T cells were electroporated with EZH2-siRNA or negative control siRNA (120 nM, GenePharma), or EZH2 plasmid or empty vector (40 ng, Genebio), or STAT3-siRNA or negative control siRNA (120 nM, GenePharma), using the 4D-Nucleofector System (Lonza). The siRNA sequences are shown in **Table S2**. Cells were rested in RPMI-1640 medium and supplemented with 10% FBS overnight. The RNA and protein levels of knockdown and overexpression efficiencies were examined at 24 and 48 h, respectively.

### Apoptosis and Proliferation Assays

Naïve CD4^+^ T cells were incubated under Tfh conditions for three days. Cells were stained with Annexin-V and 7-AAD (BD Biosciences) for 15 min at room temperature to examine apoptosis. Naïve CD4^+^ T cells were labeled with CFSE (2 µM, BD Biosciences) for 15 min followed by quenching on ice for 2 min, then were incubated under Tfh conditions and were measured for cell proliferation on day 3.

### Flow Cytometry

The following fluorochrome-conjugated antibodies were used: CD4 (RPA-T4, Biolegend), CD8 (SK1, Biolegend), CD19 (HIB19, Biolegend), CD69 (FN50, Biolegend), CD25 (BC96, Biolegend), CXCR5 (J252D4, Biolegend), PD-1 (EH12.2H7, Biolegend), IFNγ (4S.B3, Biolegend), IL-4 (MP4-25D2, Biolegend), IL-17A (BL168, Biolegend), FOXP3 (PCH101, Invitrogen), and EZH2 (11/EZH2, BD Biosciences). For surface staining, cells were stained with antibodies at 4 °C in the dark for 30 min. For intracellular cytokine staining, cells were stimulated with a leukocyte activation cocktail containing GolgiPlug (BD Bioscience) for 4 h, then fixed and permeabilized using Cytofix/Cytoperm (BD Bioscience), followed by incubation with antibodies. For FOXP3 and EZH2 staining, cells were fixed and permeabilized using the FOXP3 staining kit (Thermo Fisher) according to the instructions of the manufacturer. Cells were analyzed with a BD Aria II Flow Cytometer (BD Bioscience) and data were processed using FlowJo X (Tree Star).

### Quantitative RT-PCR

Total RNA was extracted using the RNA-Quick Purification Kit (Yishan Biotechnology) and quantified using NanoDrop2000 (NanoDrop Technologies). cDNA was synthesized with the PrimeScript RT Master Mix (Takara) and real-time PCR was performed with TB Green Premix Ex Taq II (Takara) and a Roche 480 II thermocycler. The relative expression of EZH2 and STAT3 against GAPDH was calculated using the comparative ΔΔCT method. The primer sequences are listed in **Table S2**.

### Western Blot

Total proteins were extracted from lysed cells using the Minute Total Protein Extraction Kit (Invent Biotechnologies) and were quantified using the BCA Assay kit (Pierce). Total proteins were separated by 10% sodium dodecyl sulfate-polyacrylamide gel electrophoresis, transferred to polyvinylidene fluoride membrane (Millipore), blocked with QuickBlock Western Blocking Buffer (Beyotime), incubated with anti-EZH2 (1:1000, Cell Signaling Technology), anti-pSTAT3 (1:1,000, Cell Signaling Technology), anti-STAT3 (1:1,000, Cell Signaling Technology) or anti-β-actin (1:1,000, Easybio) antibodies at 4°C overnight, then were incubated with HRP-conjugated antibody for 1 h at room temperature. Bands were detected by the Tanon 5800 Gel imaging system (Tanon). Densitometry quantification was analyzed using Image J (National Institutes of Health).

### Immunohistochemistry Assay

Labial salivary gland tissues were paraffin-embedded and cut into 3 um sections after fixation. The sections were deparaffinized, rehydrated, and heated for antigen retrieval. Endogenous peroxidase activity was blocked using 3% hydrogen peroxide. The sections were incubated overnight at 4°C with anti-EZH2 (1:200, Bioss), then incubated with a conjugated secondary antibody for 50 min at room temperature, and stained with DAB to perform a chromogenic reaction. Sections were immersed in 3,3-diaminobenzidine, counterstained with 10% Mayer’s hematoxylin, dehydrated, and mounted. The images were taken with a Nikon microscope. Quantification of immunohistochemistry staining was performed using Image J (National Institutes of Health).

### Statistical Analysis

Data were expressed as mean ± SD or median and interquartile range (IQR). Differences between the two groups were compared using unpaired *t*-test or paired *t*-test for normally distributed data or unpaired Mann–Whitney U tests or paired Wilcoxon signed-rank tests for non-normally distributed data according to Shapiro–Wilk test. Pearson’s correlation test and two-way ANOVA were applied as indicated. A two-sided *p*-value of <0.05 was considered statistically significant. All statistical analysis was performed using SPSS 25.0 software (IBM).

## Results

### Overexpressed EZH2 in CD4^+^ and CD8^+^ T Cells From pSS Patients

**Figure 1:**
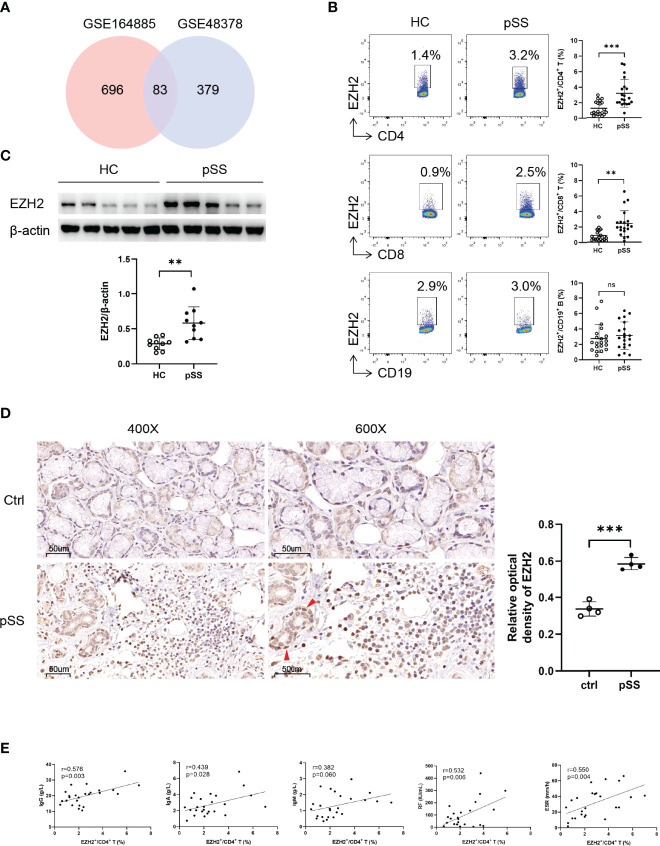
Upregulated EZH2 in pSS CD4^+^ T and CD8^+^ T cells. **(A)** Identification of highly expressed genes in peripheral blood mononuclear cells of pSS patients. The upregulated genes were defined as fold change of >1.5 and p-value of <0.05 compared with healthy controls. Eighty-three genes including EZH2 were upregulated both in GSE164885 and GSE48378 datasets. **(B)** EZH2 expression in circulating CD4^+^ T cells, CD8^+^ T cells, and CD19^+^ B cells from healthy controls (n = 20) and pSS patients (n = 20). **(C)** Western blot analysis of EZH2 expression in CD4^+^ T cells from healthy controls (n = 10) and pSS patients (n = 10). **(D)** Representative immunohistological staining of EZH2 in small labial gland from non-SS xerostomia controls (n = 4) and pSS patients (n = 4) (left, ×400; right, ×600). Positive epithelial cells were indicated using red arrows. **(E)** Correlation analysis of EZH2 expression in pSS CD4^+^ T cells and IgG (n = 25), IgA (n = 25), IgM (n = 25), RF (n = 25), and ESR (n = 25). Data were presented as mean ± SD and were obtained from independent experiments. **p <0.01, ***p <0.001 by unpaired Student’s *t*-test or Mann–Whitney U tests. Correlations were calculated using Pearson’s correlation analysis. ESR, erythrocyte sedimentation rate; HC, healthy control; IgA, immunoglobulin A; IgG, immunoglobulin G; IgM, immunoglobulin M; pSS, primary Sjögren’s syndrome; RF, rheumatoid factor. ns, not statistically significant.

To explore the EZH2 expression in pSS patients, we first analyzed two transcriptomic datasets (GSE164885 and GSE48378) of PBMCs from pSS patients and HCs. The results showed that EZH2 was upregulated in both datasets (**Figure 1A** and **Figure S1**). We then examined the EZH2 expression in circulating CD4^+^ T cells, CD8^+^ T cells, and CD19^+^ B cells from pSS patients and HCs using flow cytometry (**Figure S2**). EZH2 expression was higher in CD4^+^ T cells and CD8^+^ T cells but not in CD19^+^ B cells from pSS than in healthy controls (**Figure 1B**). Western blotting analysis confirmed the higher EZH2 expression in circulating CD4^+^ T cells from pSS (**Figure 1C**). We also examined the EZH2 expression in the salivary gland from pSS and non-SS xerostomia controls using immunohistochemistry, which revealed that EZH2 was expressed in salivary gland epithelial cells from pSS and non-SS xerostomia controls as well as in lymphocytes from pSS (**Figure 1D**). Additionally, EZH2 expression in circulating CD4^+^ T cells from pSS patients was positively correlated with serum IgG (*r* = 0.576, p = 0.003), IgA (r = 0.439, p = 0.028), RF (r = 0.532, p = 0.006) and ESR (r = 0.550, p = 0.004) (**Figure 1E**). These data suggested that circulating CD4^+^ T cells from pSS patients expressed a higher level of EZH2, which was closely correlated with B-cell hyperactivation.

### EZH2 Regulates CD4^+^ T-Cell Activation and Differentiation in pSS Patients

**Figure 2:**
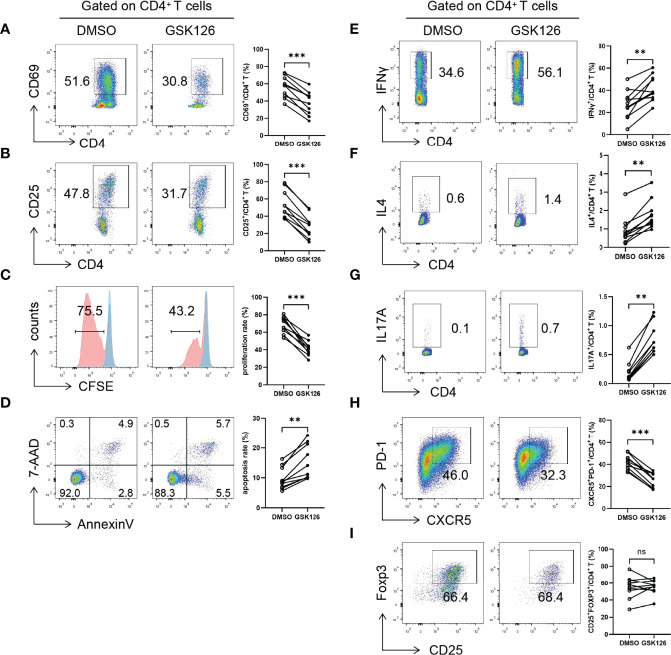
EZH2 inhibition attenuates T-cell activation and Tfh differentiation. Naïve CD4^+^ T cells from pSS patients were treated with GSK126 or DMSO, and were stimulated with anti-CD3 and anti-CD28 under Tfh condition, and **(A)** CD69 expression (n = 10), **(B)** CD25 expression (n = 10), **(C)** proliferation (n = 10), and **(D)** apoptosis (n = 10) were measured on day 3. Naïve CD4^+^ T cells from pSS patients were treated with GSK126 or DMSO, and were stimulated with anti-CD3 and anti-CD28 under Th1, Th2, Th17, Tfh, and Treg-polarizing conditions, respectively, and **(E)** IFNγ^+^ Th1 (n = 10), **(F)** IL4^+^ Th2 (n = 10), **(G)** IL17A^+^ Th17 (n = 10), **(H)** CXCR5^+^PD-1^+^ Tfh (n = 10), **(I)** CD25^hi^FOXP3^+^ Treg cells (n = 10) was measured on day 5, respectively. Data were presented as mean ± SD and were obtained from independent experiments. **p <0.01, ***p <0.001 by paired Student’s *t*-test or Wilcoxon signed-rank tests. ns, not statistically significant.

To elucidate the potential function of EZH2 in pSS CD4^+^ T cells, we pretreated naïve CD4^+^ T cells from pSS patients with GSK126, a highly-selective EZH2 inhibitor, or DMSO and examined the activation, proliferation, apoptosis, and differentiation of CD4^+^ T cells (**Figure S3**). GSK126 suppressed CD69 (**Figure 2A**) and CD25 (**Figure 2B**) expression and proliferation (**Figure 2C**) and promoted apoptosis of CD4^+^ T cells (**Figure 2D**) under Tfh conditions. Notably, GSK126 promoted IFNγ + Th1 (**Figure 2E**), IL4 + Th2 (Figure 2F), and IL17A + Th17 (**Figure 2G**), attenuated CXCR5+PD-1 +Tfh differentiation (**Figure 2H**), but not CD25hiFOXP3 +Treg differentiation (**Figure 2I**). Together, EZH2 inhibition with GSK126 attenuated the activation, proliferation, and Tfh differentiation of CD4^+^ T cells, suggesting that overexpressed EZH2 was implicated in regulating CD4^+^ T cells in pSS.

### EZH2 Promotes Tfh Differentiation

**Figure 3:**
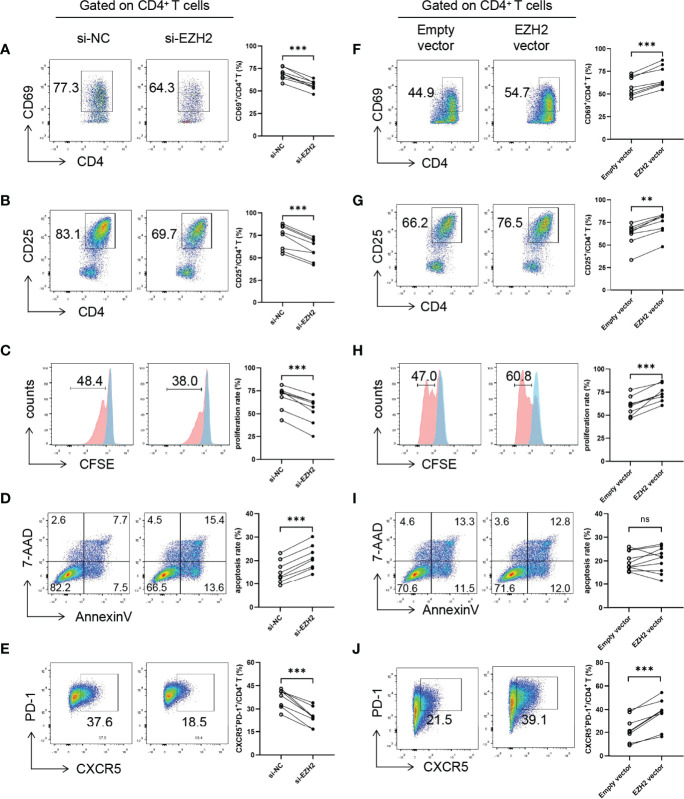
EZH2 promotes Tfh differentiation. Naïve CD4^+^ T cells from healthy controls were transfected with negative control (NC) or EZH2 siRNA and were incubated under Tfh condition, **(A)** CD69 expression (n = 8), **(B)** CD25 expression (n = 8), **(C)** proliferation (n = 8), and **(D)** apoptosis (n = 8) were measured on day 3, and **(E)** CXCR5^+^PD-1^+^ Tfh cells (n = 8) were measured on day 5. Naïve CD4^+^ T cells from healthy controls were transfected with empty or EZH2-expressing vector and were incubated under Tfh condition, **(F)** CD69 expression (n = 8), **(G)** CD25 expression (n = 8), **(H)** proliferation (n = 8), and **(I)** apoptosis (n = 10) were measured on day 3, and **(J)** CXCR5^+^PD-1^+^ Tfh cells (n = 8) were measured on day 5. Data were presented as mean ± SD and were obtained from independent experiments. **p <0.01, ***p <0.001 by paired Student’s *t*-test or Wilcoxon signed-rank tests. Ns, not statistically significant. NC, negative control.

Since Tfh cells play an essential role in pSS and EZH2 expression in circulating CD4^+^T cells was positively correlated with B-cell hyperactivation and the circulating Tfh population in pSS (**Figure S4**), we focused on the role of EZH2 in Tfh differentiation. We downregulated and upregulated EZH2 in naïve CD4^+^ T cells from healthy controls using siRNAs or plasmids, which was confirmed by quantitative PCR and Western blotting (**Figures S5A–D**). EZH2 knockdown resulted in lower levels of CD69 and CD25 (**Figures 3A, B**), lower proliferation (**Figure 3C**), higher apoptosis (**Figure 3D**), and attenuated Tfh differentiation (**Figure 3E**), which were consistent with GSK126 inhibition. In contrast, EZH2 overexpression promoted CD69 and CD25 expression (**Figures 3F, G**), and proliferation (**Figure 3H**), but not apoptosis (**Figure 3I**). Besides, EZH2 overexpression promoted Tfh differentiation (**Figure 3J**). Therefore, these data further suggest that EZH2 promotes the activation, proliferation, and differentiation of Tfh cells.

### EZH2 Promotes Tfh Differentiation Through Enhancing STAT3 Phosphorylation

**Figure 4:**
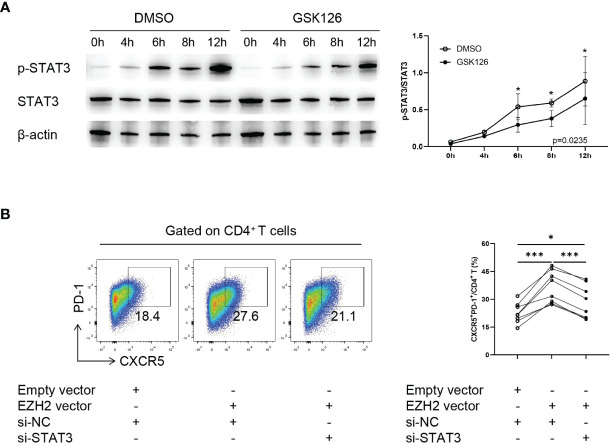
EZH2 promotes Tfh differentiation through enhancing STAT3 phosphorylation. **(A)** Representative Western blot of and summary statistics of phosphorylated-STAT3 (p-STAT3) and STAT3 in naïve CD4^+^ T cells from healthy controls treated with DMSO or GSK126 at 0, 4, 6, 8, and 12 h under Tfh condition (n = 3). *P-*value was calculated using two-way ANOVA. **(B)** Naïve CD4^+^ T cells from healthy controls were transfected with control siRNA, STAT3 siRNA, EZH2 vector, and (or) empty vector and were incubated under Tfh condition, and CXCR5^+^PD-1^+^ Tfh cells (n = 8) were measured on day 5. Data were presented as mean ± SD and were obtained from independent experiments. *p <0.05, ***p <0.001 by paired Student’s *t*-test or Wilcoxon signed-rank tests. NC, negative control.

Finally we explored the underlying mechanism of EZH2-mediated Tfh differentiation. Given that EZH2 interacts with STAT3, a critical transcription factor of Tfh differentiation and enhances its phosphorylation (24, 26), we hypothesized that EZH2 might promote Tfh differentiation through STAT3. We observed that GSK126 significantly inhibited STAT3 phosphorylation in CD4^+^ T cells without modulating STAT3 expression (**Figure 4A**).

To confirm that EZH2 regulated Tfh differentiation in a STAT3-dependent manner, we downregulated STAT3 using siRNA (**Figures S6A, B**) in EZH2-overexpressed naïve CD4^+^ T cells. Downregulating STAT3 significantly suppressed Tfh differentiation induced by EZH2 overexpression (**Figure 4B**). Collectively, these data suggest that EZH2 promotes Tfh differentiation by enhancing STAT3 phosphorylation.

## Discussion

In this study, we investigated the potential role of EZH2 in pSS. EZH2 was aberrantly overexpressed in pSS CD4^+^ T cells and was positively correlated with B-cell hyperactivation markers and the circulating Tfh population. Importantly, EZH2 promoted the activation, proliferation, and Tfh differentiation of CD4^+^ T cells. Mechanistically, EZH2 enhances STAT3 phosphorylation to promote Tfh differentiation. Overall, our study suggests a role for EZH2 in pSS pathogenesis.

EZH2 regulates the development of T cells (27). Specifically, EZH2 plays a versatile role in the differentiation and plasticity of CD4^+^ T cells. Several studies suggest a repressive role of EZH2 in the differentiation of Th1, Th2, and Th17 cells, which is consistent with our findings. EZH2-deficient CD4^+^ T cells enhance IFN-γ, IL-4, and IL-17 production, and T-bet, Gata-3 expression (28–30). However, previous studies indicate that EZH2 might promote Th1 and Th2 polarization (31, 32), suggesting that the role of EZH2 in Th1 and Th2 differentiation is controversial. EZH2 is crucial in maintaining Treg function and differentiation (28, 33). However, we did not observe a significant change in Treg differentiation using EZH2 inhibition. EZH2 promotes the polarization of mouse Tfh cells during viral infection (34, 35). EZH2 deficiency in a lupus-like chronic graft versus host disease mouse model significantly reduces Tfh cells and suppresses antibody production and germinal center formation (36). Our findings highlighted that EZH2 promoted Tfh differentiation in pSS, which participates in the pathogenesis of pSS. EZH2 expression was correlated with immunoglobin level and the circulating Tfh population, suggesting that EZH2 overexpression might induce Tfh expansion in pSS patients and subsequently promote B cell hyperactivation. Therefore, targeting EZH2 might be a theoretical approach for Tfh overactivation in pSS.

EZH2 regulates Tfh differentiation through several mechanisms. EZH2 deploys H3K27me3 to repress Cdkn2a expression, a key suppressor of Bcl6, thus enhancing Tfh differentiation (34). Besides, EZH2 PRC2-independently acts as a transcriptional coactivator (37). EZH2 cooperates with Tcf1 to activate Bcl6 transcription, which requires the phosphorylation of EZH2 (34). In addition, EZH2 facilitates chromatin accessibility of Tfh-signature genes, including Bcl6, at early Tfh fate commitment (35). EZH2 also methylates non-histone proteins (38), such as STAT3. EZH2 binds and methylates STAT3 at K180 and enhances STAT3 tyrosine phosphorylation in glioblastoma, possibly by protecting it from dephosphorylation (26). EZH2 methylates and enhances STAT3 phosphorylation in keratinocytes and promotes inflammation in the psoriasis model (24). STAT3 is a critical transcription factor for Tfh differentiation and is activated by IL6, IL12, IL21, IL23, and TGF-β (39). Autosomal-dominant hyper-IgE syndrome patients carrying STAT3 mutations have lower circulating Tfh cells with B-cell-helping function defects (40), and STAT3-deficient mice show defective Tfh and GC-B cells (41). STAT3 binds the Bcl6 locus in the presence of IL6 (42) and promotes Bcl6 expression with the Ikaros zinc finger transcription factors Aiolos and Ikaros (43). We found that EZH2 also enhanced STAT3 phosphorylation in CD4^+^ T cells and promoted Tfh cell differentiation. Therefore, we inferred a new mechanism of EZH2-mediated Tfh differentiation in pSS, which was enhanced STAT3 phosphorylation and potential methylation of STAT3.

The therapeutic potential of EZH2 inhibitors has been shown in autoimmune diseases. Inhibiting EZH2 in NZB/NZW F1 lupus mice reduces anti-dsDNA autoantibodies, improves nephritis, and prolongs survival by blocking the IFN-I signaling pathway (44). EZH2 depletion in experimental autoimmune encephalomyelitis attenuates inflammation by activating SOCS3 (45). EZH2 inhibition effectively attenuates psoriasis-like skin lesions by suppressing STAT3-mediated IκBζ expression (24). Consistently, we found that inhibiting EZH2 using GSK126 leads to decreased Tfh differentiation. However, we did not examine the therapeutic potential of EZH2 inhibitor in the pSS model *in vivo*, which was a limitation of our study and warrants further study in the experimental Sjögren’s syndrome model.

In summary, we demonstrated that pSS CD4^+^ T cells aberrantly overexpressed EZH2, which promoted activation, proliferation, and Tfh cell differentiation by enhancing STAT3 phosphorylation. Our findings indicate that EZH2 could promote Tfh differentiation and might be implicated in pSS pathogenesis.

## Data Availability Statement

The datasets presented in this study can be found in online repositories. The names of the repository/repositories and accession number(s) can be found in the article/**Supplementary Material**.

## Ethics Statement

This study was approved by the Ethics Committee of PUMCH (Approval number: S-K2023). The patients/participants provided their written informed consent to participate in this study.

## Author Contributions

CH designed the study, performed the experiment, and wrote the manuscript. YY, ZC, SL, TL, and LZ collected samples and performed data analysis. LW, YL, MW recruited patients. HC and FZ directed the study and revised the manuscript. All authors listed have made a substantial, direct, and intellectual contribution to the work and approved it for publication.

## Funding

This study was supported by the National Key R&D Program of China (2016YFA0101003, 2016YFC0903901, 2018YFE0207300), the National Program of Key Research Project of New Drug Innovation (2008ZX09312-016), the National Natural Science Fund (82071842, 81771764), and the CAMS Innovation Fund for Medical Sciences (2017-I2M-3-007).

## Conflict of Interest

The authors declare that the research was conducted in the absence of any commercial or financial relationships that could be construed as a potential conflict of interest.

## Publisher’s Note

All claims expressed in this article are solely those of the authors and do not necessarily represent those of their affiliated organizations, or those of the publisher, the editors and the reviewers. Any product that may be evaluated in this article, or claim that may be made by its manufacturer, is not guaranteed or endorsed by the publisher.
