# Supplementary figures and images for: EZH2 Promotes T Follicular Helper Cell Differentiation Through Enhancing STAT3 Phosphorylation in Patients With Primary Sjögren’s Syndrome
Source: Front Immunol. 2022 Jun 20;13:922871. doi: 10.3389/fimmu.2022.922871 (PMC9252457; doi:10.3389/fimmu.2022.922871)

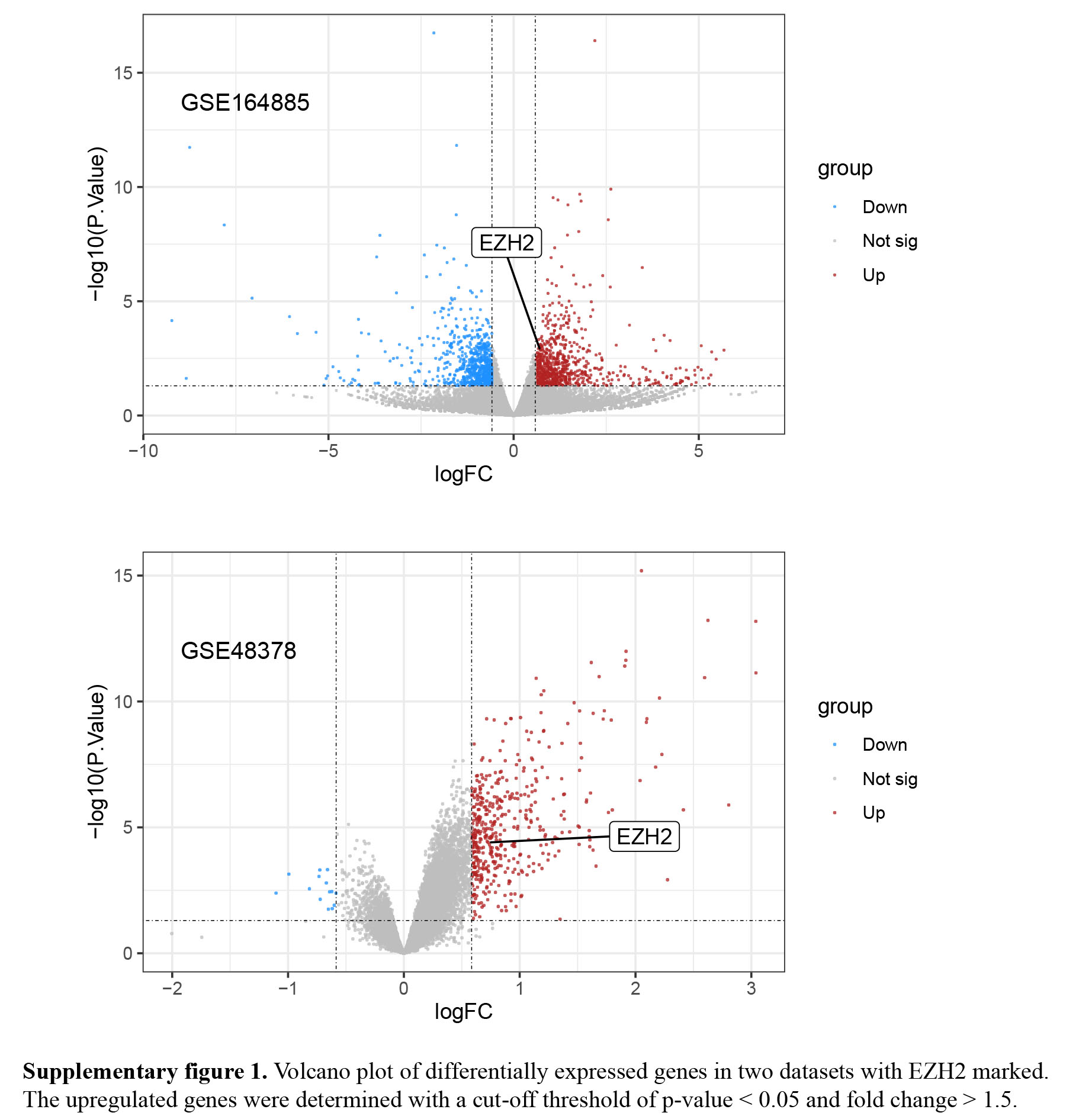

Supplement: Supplementary file 1 [file Image_1.jpeg]

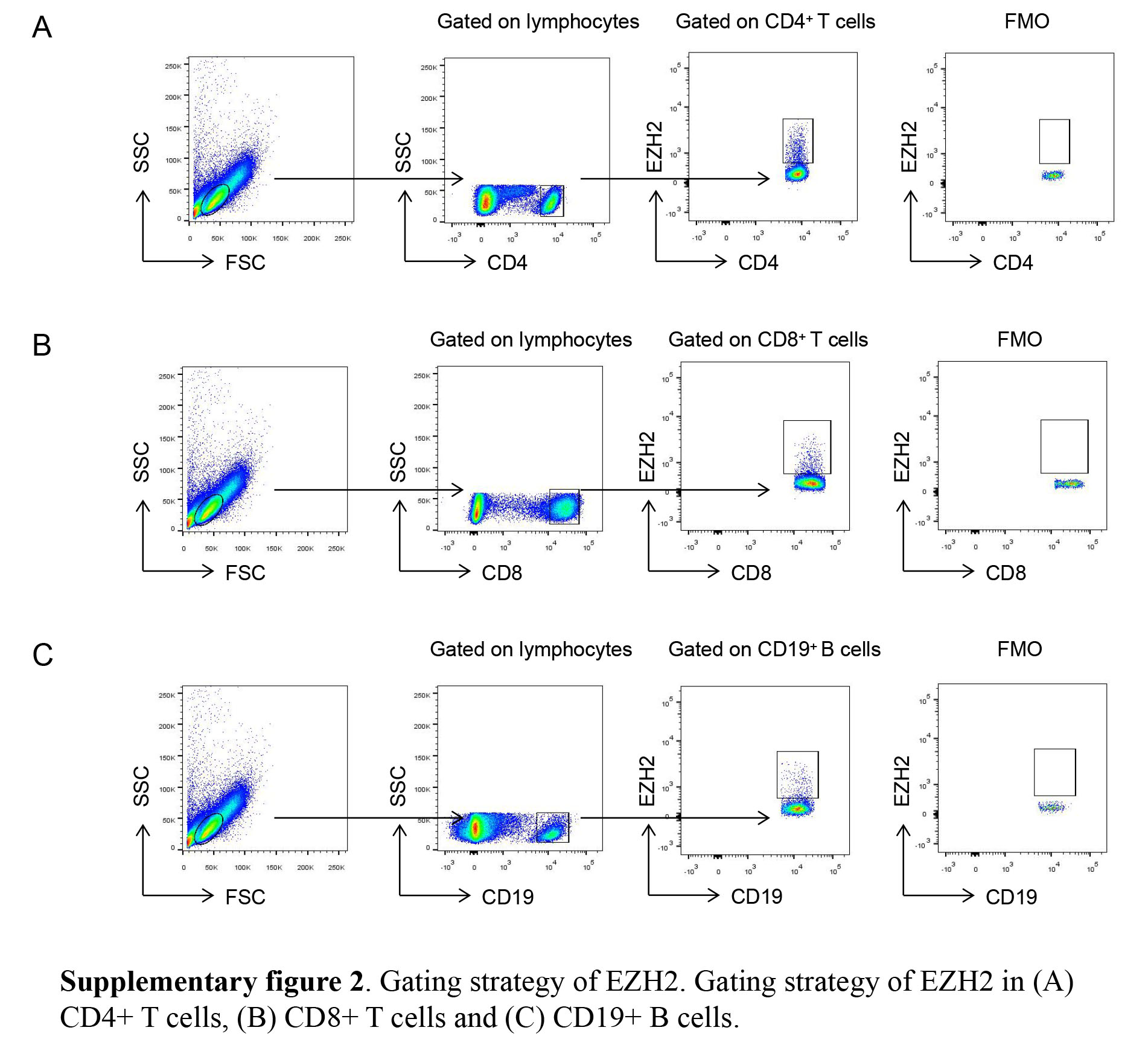

Supplement: Supplementary file 2 [file Image_2.jpeg]

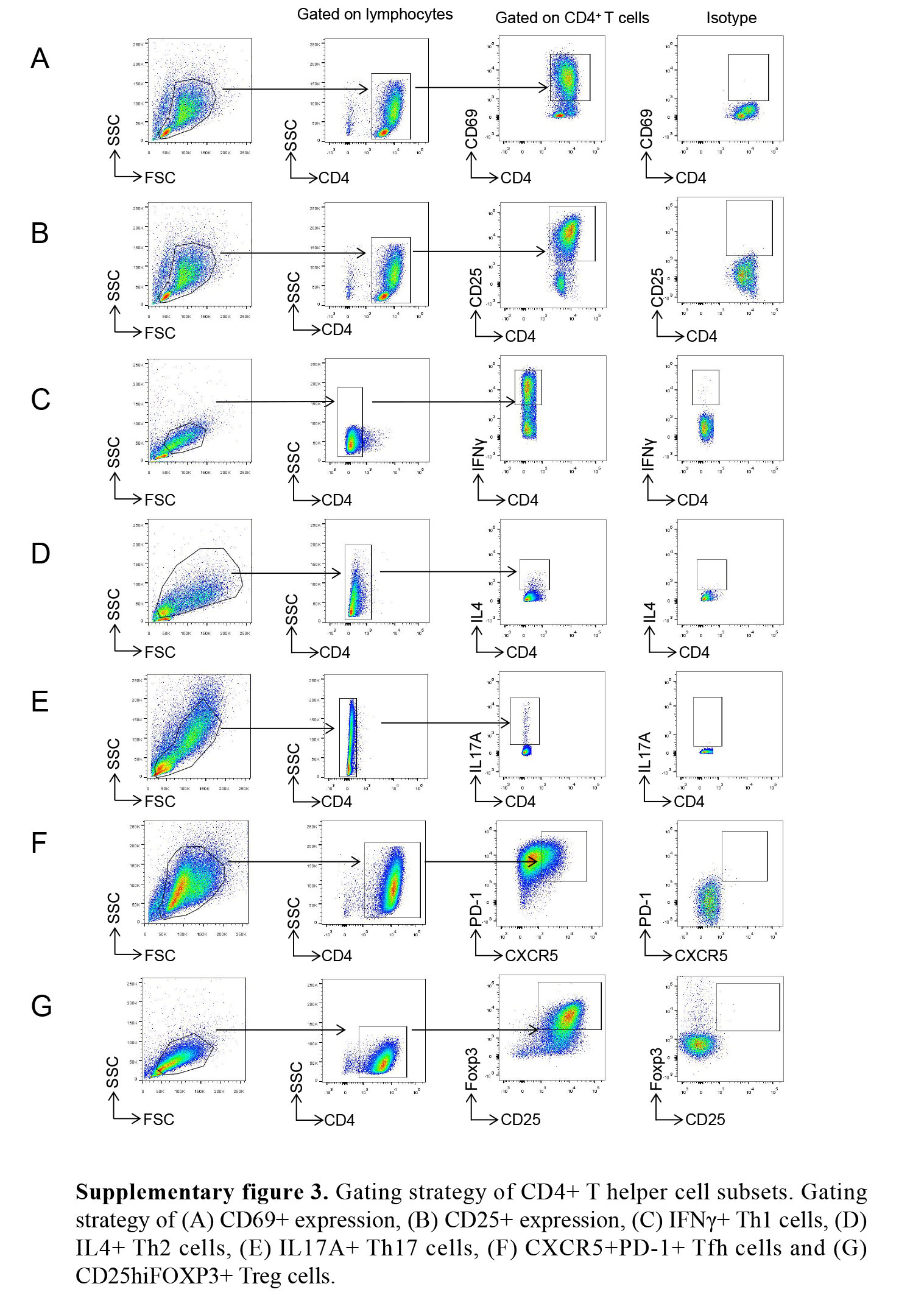

Supplement: Supplementary file 3 [file Image_3.jpeg]

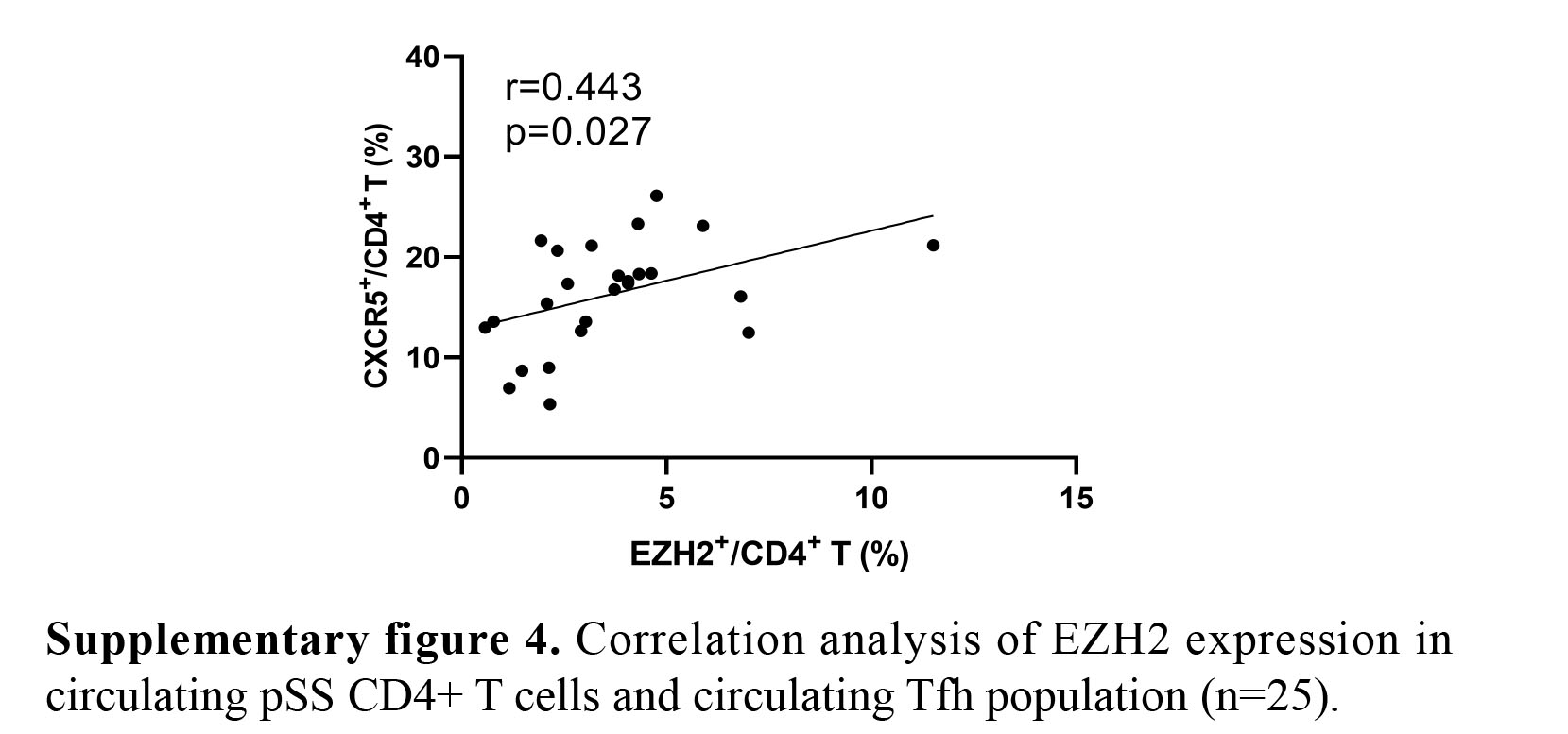

Supplement: Supplementary file 4 [file Image_4.jpeg]

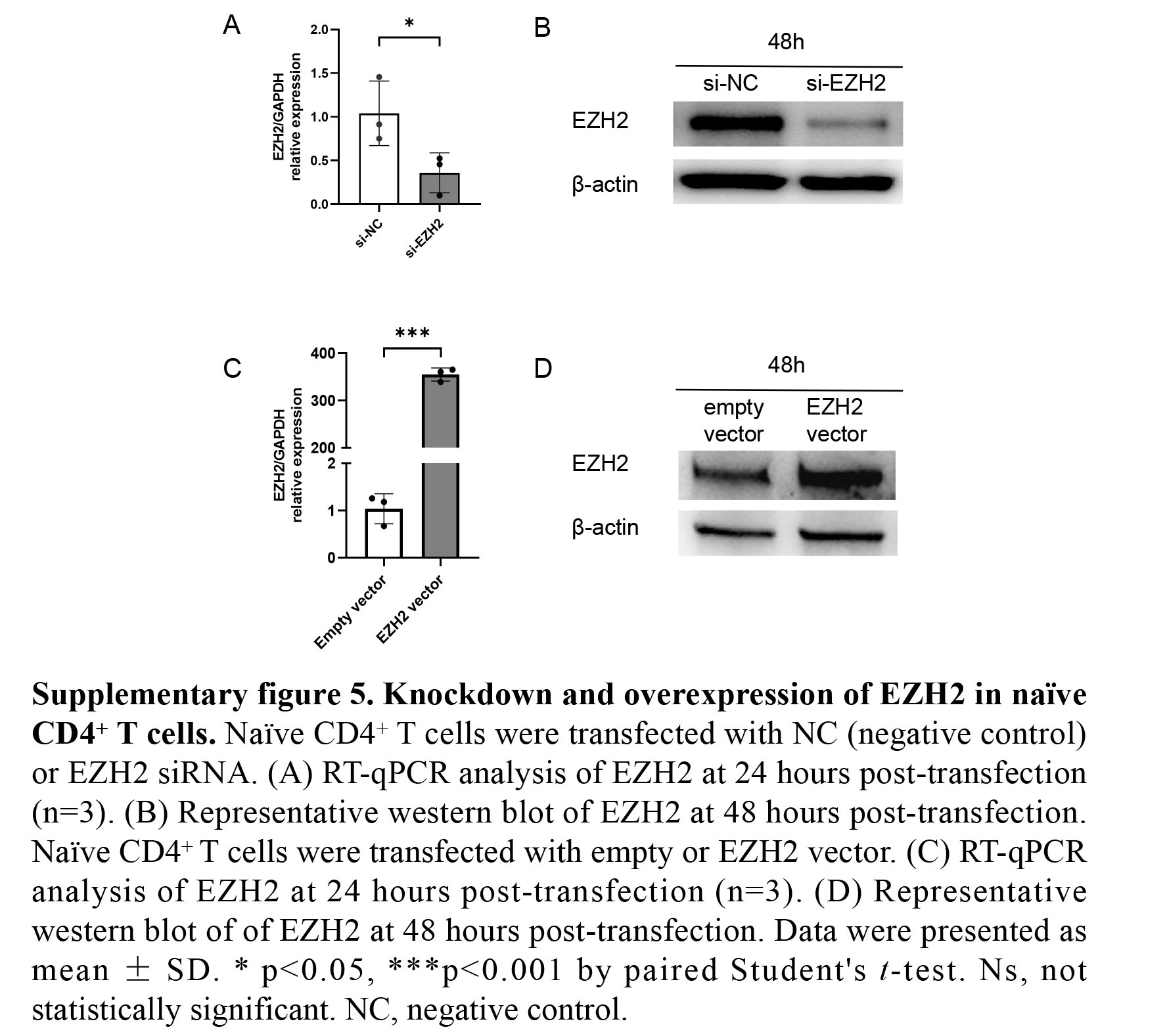

Supplement: Supplementary file 5 [file Image_5.jpg]

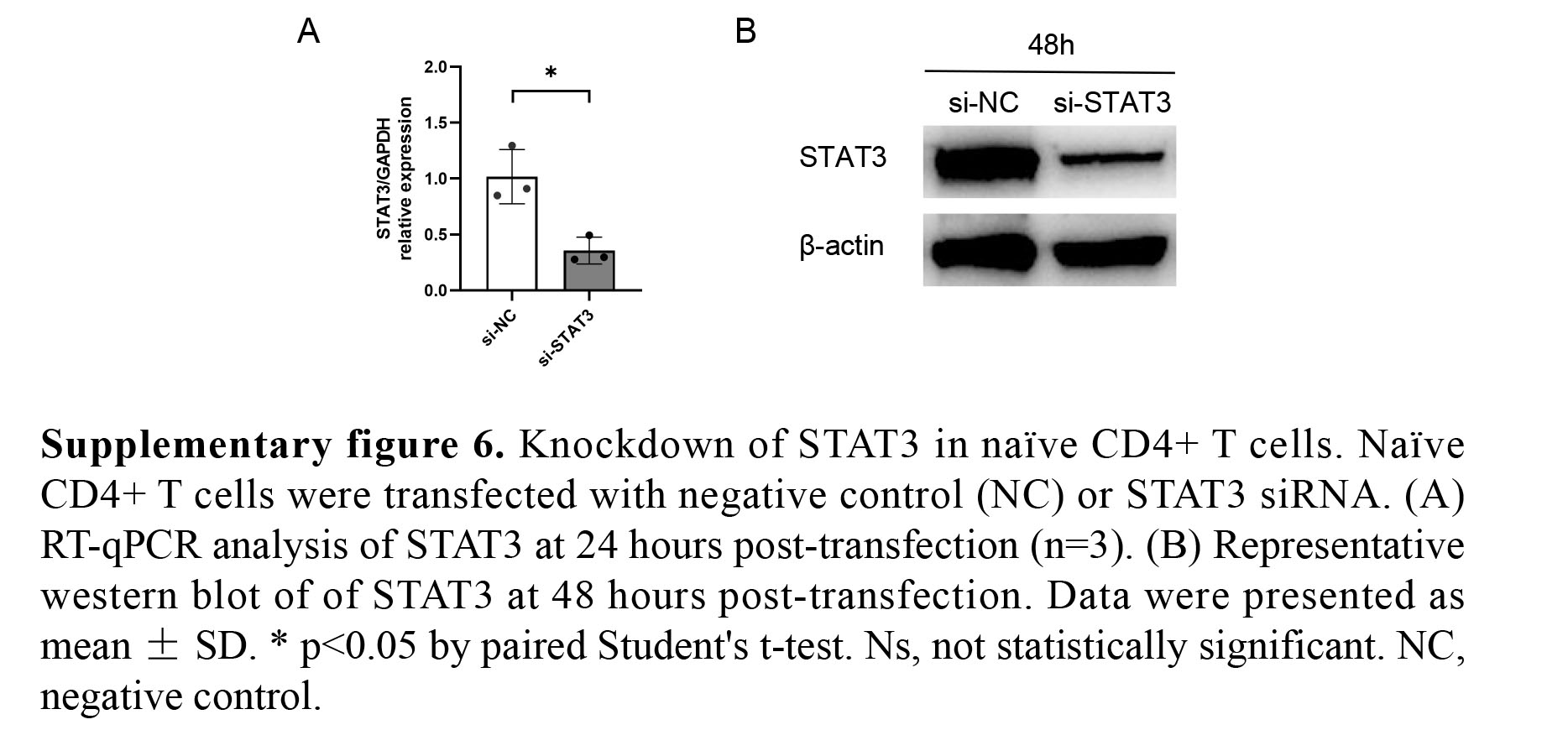

Supplement: Supplementary file 6 [file Image_6.jpg]
