# Supplementary material for: EZH2 Promotes T Follicular Helper Cell Differentiation Through Enhancing STAT3 Phosphorylation in Patients With Primary Sjögren’s Syndrome
Source: Front Immunol. 2022 Jun 20;13:922871. doi: 10.3389/fimmu.2022.922871 (PMC9252457; doi:10.3389/fimmu.2022.922871)
Supplement: Supplementary file 7 [file Table_1.docx]

**Supplementary table 1.** Clinical characteristics of pSS patients in the study.

|  | **pSS patients (n=127)** |
| --- | --- |
| Age (years) | 50.0 (38.0-57.0) |
| Female sex, n (%) | 127 (100%) |
| **Clinical features** |  |
| Dry mouth, n (%) | 93 (73.2%) |
| Dry eyes, n (%) | 69 (54.3%) |
| Parotid swollen, n (%) | 14 (11.0%) |
| Xerophthalmia, n (%) | 60 (47.2%) |
| Focus score ≥1, n (%) | # |
| Low salivary flow rate, n (%) | 72 (56.7%) |
| **Laboratory features** |  |
| ANA positive, n (%) | 112 (88.2%) |
| anti-SSA positive, n (%) | 111 (87.4%) |
| anti-SSB positive, n (%) | 57 (44.9%) |
| IgG (g/L) | 16.9 (13.3-21.1) |
| RF (U/L) | 42.5 (17.1-111.0) |
| ESR (mm/h) | 17.0 (11.0-32.5) |
| **Extra glandular involvement** |  |
| ILD, n (%) | 13 (10.2%) |
| Arthritis, n (%) | 10 (7.9%) |
| Renal involvement, n (%) | 6 (4.7%) |
| Cutaneous involvement, n (%) | 7 (5.5%) |
| Peripheral neuropathy, n (%) | 2 (1.6%) |
| Haematological involvement, n (%) | 5 (3.9%) |
| **Treatment** |  |
| Hydroxychloroquine, n (%) | 85 (66.9%) |
| Glucocorticoids, n (%) | 48 (37.8%) |
| Immunosuppressants, n (%) | 37 (29.1%) |

# Only four patients received labial salivary gland biopsy with a focus score ≥1, and immunohistochemistry results in figure 1D were obtained from these patients. Quantitative data were expressed as medians with interquartile ranges. Categorical variables were expressed as counts and percentages. ANA, antinuclear antibody; Anti-SSA, Anti-Ro/Sjögren's syndrome A antigen; Anti-SSB, Anti-La/Sjögren's syndrome B antigen (SSB); ESR, erythrocyte sedimentation rate; IgG, Immunoglobulin G; ILD, interstitial lung disease; RF, rheumatoid factor. NA, not available.
