# Supplementary material for: EZH2 Promotes T Follicular Helper Cell Differentiation Through Enhancing STAT3 Phosphorylation in Patients With Primary Sjögren’s Syndrome
Source: Front Immunol. 2022 Jun 20;13:922871. doi: 10.3389/fimmu.2022.922871 (PMC9252457; doi:10.3389/fimmu.2022.922871)
Supplement: Supplementary file 8 [file Table_2.docx]

**Supplementary table 2**. Primer and siRNA sequences.

| Gene | Sequence (5'-3') |
| --- | --- |
| EZH2 primer forward | TTCATGCAACACCCAACACT |
| EZH2 primer reverse | GGTGGGGTCTTTATCCGCTC |
| STAT3 primer forward | CAGCAGCTTGACACACGGTA |
| STAT3 primer reverse | AAACACCAAAGTGGCATGTGA |
| GAPDH primer forward | TCAACGACCACTTTGTCAAGCTCA |
| GAPDH primer reverse | GCTGGTGGTCCAGGGGTCTTACT |
| EZH2 siRNA sense | CCUGACCUCUGUCUUACUUTT |
| EZH2 siRNA antisense | AAGUAAGACAGAGGUCAGGTT |
| STAT3 siRNA sense | GCUGAACAACAUGUCAUUUTT |
| STAT3 siRNA antisense | AAAUGACAUGUUGUUCAGCTT |
